# Consumption of Native Fish Associated with a Potential Carcinogenic Risk for Indigenous Communities in the Peruvian Amazon

**DOI:** 10.3390/toxics12080552

**Published:** 2024-07-30

**Authors:** Magaly Alejandra Brousett-Minaya, Fred William Chu-Koo, Juvenal Napuchi-Linares, Cynthia Elizabeth Zambrano Panduro, Juan Amilcar Reyes-Larico, Adriana Edith Larrea-Valdivia, Ivan Edward Biamont-Rojas

**Affiliations:** 1 Faculty of Sciences, National Autonomous University of Alto Amazonas (UNAAA), Prolongación Libertad 1220-1228, Yurimaguas 16501, Loreto, Peru; fchu@unaaa.edu.pe (F.W.C.-K.); jnapuchi@unaaa.edu.pe (J.N.-L.); 1261903137@unaaa.edu.pe (C.E.Z.P.); 2 Faculty of Natural Sciences, National University of San Agustin—Arequipa (UNSA), Santa Catalina No. 117, Arequipa 04000, Arequipa, Peru; jreyesl@unsa.edu.pe (J.A.R.-L.);; 3 Institute of Science and Technology, State University of São Paulo (UNESP), Av. Três de Março 511, Alto da Boa Vista, Sorocaba 18087-180, SP, Brazil; biamont.ivan@gmail.com; 4 Oceanographic Institute, University of São Paulo (USP), Praça do Oceanográfico, 191, São Paulo 05508-120, SP, Brazil

**Keywords:** human health risk assessment, fluvial sediment, fish muscle, carcinogenic index, heavy metals

## Abstract

Aquatic environments, such as fluvial environments, play an important role in the transport of material from throughout the basin, and this material partially sediments along the way. The objective of this study was to analyze, from an ecotoxicological point of view, the concentrations of arsenic and heavy metals in sediment and the muscle of native fish, to correlate their interaction and to evaluate the potential risk to public health using carcinogenic risk indices in four rivers of the Peruvian Amazon. There were 27 sampling sites where sediment and fish (except for five points) samples were collected. A sampling pool was created with fish muscles from all species collected at each sampling site. Concentrations of As, Cd, Cr, Cu, Ni, Pb, Zn, and Hg were analyzed in both sediment and fish muscle, in duplicate. The results indicate the presence of concentrations higher than those recommended by international guidelines for sediment and food. Mercury (Hg) concentrations in the Tigre, Morona, and Pastaza rivers are up to six times higher than the recommended value for daily consumption. The carcinogenic risk due to the regular consumption of native species in the indigenous communities living on the banks of the four studied rivers is high.

## 1. Introduction

The minerals found in the basins are transported to water bodies due to natural and artificial actions. Some of these elements are essential for the dynamics in aquatic environments, and for life itself [1,2]. A significant part of the rivers in the Amazon basin originates in the Peruvian and Ecuadorian Andes, draining their waters through steep and rocky volcanic terrain, carrying metals or soluble chemical compounds in their path [3]. Minerals, because of their polar properties, are adsorbed to particles (e.g., organic matter, dead cells, etc.), making them heavier and causing them to sediment [4,5,6]. This results in a natural sink for all of the material derived from natural or anthropic sources in the basin [7]. The sedimented material can easily be resuspended due to physical phenomena (e.g., wind, precipitation, current alteration, etc.), which increases their negative impact on the aquatic environment [2].

When metals and metalloids, as part of the minerals, are found in high concentrations in different environmental compartments, such as sediment, they become an environmental threat [8,9] to the aquatic system and to public health. The Amazon Basin is susceptible to these fluctuations, especially for human communities settled in riverside zones that culturally have a major food dependency on aquatic ecosystems for fishing and drinking water [10].

Artisanal mining is one of the main activities in the upper basin area (Peru and Ecuador). In this type of mining, chemicals such as mercury and cyanide 8 are frequently employed without supervision. Additionally, mining activity in the Ecuadorian Amazon tripled in size from 2015 to 2021 (7495 ha) across various provinces (Chinchipe, Napo, Morona Santiago, and Sucumbíos) [11,12,13,14]. The major concern is that not just mining activities but also petroleum extraction in the area jeopardizes indigenous communities in the Pastaza, Corrientes, Tigre, and Marañón rivers [15,16]. Both activities endanger the ichthyological diversity in the Amazonian rivers, considered the world’s highest diversity (2406 species) [17]. 

The persistence of minerals in aquatic bodies leads to their accumulation in living organisms. Some of these elements are difficult to metabolize and, therefore, remain in the organism, a phenomenon known as bioaccumulation. Another issue to consider is the process of accumulation within the food web itself, which can be related to each species’ feeding habits (detritivores, carnivores, benthonic, etc.) [18,19]. When some species feed on contaminated organisms, there is an increasing concentration of a particular metal or metalloid in the predator, a process known as biomagnification. These hydrobiological resources, many of which are essential in the diet of Amazonian people, are increasingly exposed to higher concentrations of elements in fluvial and lacustrine environments.

People of the Peruvian Amazon fish a wide variety of species (approximately 60 species), and fish consumption in this area is almost three times the national average, reaching 50 kg/person/year [10]. Indigenous people (105,900 inhabitants) [20] live in this area. Ethnic groups such as the Kichwa, Achuar, Wampis, Shapra, and Kandozi are mainly settled along the sides of the Pastaza, Morona, Tigre, and Corrientes rivers, with a traditional dependence on fish.

In this context, the presence of metals or metalloids in fluvial sediments, due to physical disturbances, can resuspend and increase bioaccumulation and biomagnification processes that affect fish, turning them into potential carriers of toxic substances in the food web. As far as is known, this is the first study in the Peruvian Amazon that relates the presence of metals and metalloids in fish muscles to their concentrations in fluvial sediments in areas affected by mining and petroleum activities. This study makes a significant contribution to environmental toxicology and geochemistry in Amazonian fluvial environments. Finally, the results explore the probable carcinogenic and non-carcinogenic risk occurrences due to fish consumption in human communities settled along four rivers in the Peruvian Amazon.

Therefore, the objective was to analyze the arsenic and heavy metal concentrations in both the sediment and native fish muscles from the ecotoxicological perspective, to correlate their interaction and assess their potential risk to public health, employing carcinogenic risk indices in four rivers of the Peruvian Amazon (Tigre, Morona, Corrientes, and Pastaza).

## 2. Material and Methods

During February and March 2023, sediment and fish samples were collected from four rivers in two provinces: Loreto (Corrientes and Tigre rivers) and Datém del Marañón (Pastaza and Morona rivers), both in the department of Loreto, Peru. The collections coincided with the time of high water, so all of the rivers were navigable, with considerable daily fluvial transit among rural areas where small indigenous communities are settled. These native communities are established along the riversides, with fish being their main food source.

**Figure 1:**
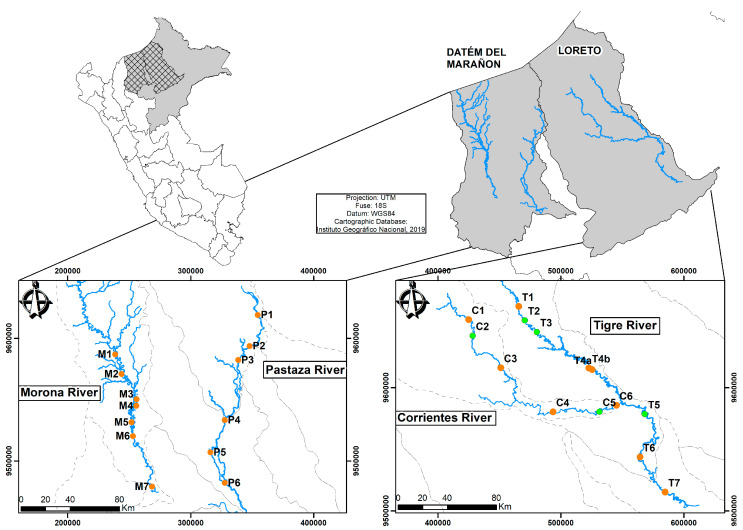
The Morona, Pastaza, Corrientes, and Tigre rivers are located in the northeast of Peru, in the department of Loreto. These rivers have their headwaters in Ecuadorian territory; from there, they flow into the Peruvian Amazon, joining the Marañón River, which, in confluence with the Ucayali River, forms the Amazon River. Sediment and fish sampling sites are in orange; sediment-only sampling sites are in green.

Sediments were collected from 27 sites along the Tigre (T1–T7), Corrientes (C1–C6), Pastaza (P1–P6), and Morona (M1–M7) rivers (Figure 1 and Table 1). Petroleum storage reservoirs were observed at some sampling sites, which belong to PetroPeru. Fish samples were collected at the same sites as the sediments, from a total of 22 sites in fishing zones, except at C2, C5, T2, T3, and T5. Sediment and fish sampling sites are represented by orange dots, while sediment-only sampling sites are represented by green dots (Figure 1).

### 2.1. Fish Sampling

Fish were collected from different fishing areas located in the four rivers. These fish included carnivorous, omnivorous, and detritivore species (Table 2). The collection followed the protocols established by the Peruvian Ministry of Environment [21]. This method involved setting up fishing nets that remained in place for 24 h. These nets were periodically checked to ensure that predator fish did not damage the captured samples, and to prevent major vertebrates (reptiles, aquatic mammals, etc.) from being accidentally trapped. A total of 404 individuals were collected from the four rivers. Approximately 500 to 800 g of fish muscle was collected at the different sampling sites, according to the number of individuals captured (Table 1).

Fish muscle extraction was performed according to the Peruvian Technical Standard 700.002 (NTP, 2021). A sampling pool was created with fish muscles from all species collected at each sampling site. Sample pooling is a method where units that were supposed to be measured individually are processed together, making separate analyses impossible, because human residents of the indigenous communities did not consume only one species of fish per day but a variety of species existing in their catches. Sampling pool analyses were carried out, since the indigenous people do not have a preference for a particular fish species, nor do they select them in their daily diet for reasons of the place they occupy in the water column or because of the fish’s diet. The sample pools were stored at −20 °C until their evaluation in the laboratory. 

### 2.2. Sediment Sampling

A total of 50 samples were collected from the Tigre (12 samples), Corrientes (12 samples), Pastaza (12 samples), and Morona (14 samples) (Figure 1). Samples were extracted from the upper layer (0–10 cm), extracted using a Van Veen sediment sampler (1000 cm^3^). The sediment was stored in plastic flasks (300 cm^3^) in the dark and kept at 4 °C until analysis in the laboratory.

### 2.3. Daily Fish Intake

To calculate the daily fish intake in riverside indigenous communities, census data from the National Institute of Statistics and Informatics [22] were used, indicating a total of 29,084 people living in the areas of interest. With this total number, and using finite population sampling (*p* = 0.05) [23], a representative sample of 1356 people from different indigenous communities was calculated and interviewed.

### 2.4. Metal Concentration Analysis

Fish muscle: The biological sample analysis was performed at the National University of San Agustín, following the method mentioned by Qin et al. (2015) [24], with partial modifications. This process took 0.5 g of the homogenized samples (epaxial muscle without skin), which were then subjected to microwave digestion in Teflon vessels (Milestone, Sorisole, Italy, Ethos Easy model). In the vessels, 10 mL of a HNO_3_:H_2_O_2_ mixture (at an 8:2 ratio) received the homogenized sample, which was heated to 280 °C in a thirty-minute ramp. The resulting sample was filtered through a PVDF membrane filter (Whatman, Maidstone, UK, 0.45 µm), and then it was diluted with Milli-Q water up to 50 mL and stored at 4 °C. Metals, except mercury, were analyzed via inductively coupled plasma mass spectrometry (ICP-MS) (PerkinElmer, Waltham, MA, USA, NexION 2000C model). Mercury was analyzed directly in an automatized mercury analyzer (Milestone, DMA-80 evo model).

Sediment: Eleven elements were studied (As, Cd, Cr, Cu, Ni, Pb, Al, Ba, Fe, Zn, and Hg). Metals, except Hg, were analyzed using inductively coupled plasma optical spectrometry (ICP-OES). This procedure requires a previous digestion of the sample, following the method of SW 846 US EPA 3050 [25]. It consisted of acid digestion, with the addition of HNO_3_, H_2_O_2_, and HCl in 4.5 h at 90 °C. In the case of mercury (Hg), it was analyzed via cold vapor atomic absorption spectroscopy (CVAAS), following the USEPA 7471B method [26], where the sample must be digested by a sequence of reactions employing H_2_SO_4_, HNO_3_, and KMnO_4_ in 2 h. Metal and metalloid data are expressed as milligrams per kilogram of dry weight (mg/kg dw).

### 2.5. QA/QC

Quality control was performed through the evaluation of the precision and accuracy of the methodology, as follows: Analytical-grade reagents (obtained from PerkinElmer) were used in all of the analyses. All glassware items and equipment used to store and process the samples for metal assessment were left in 10% nitric acid for at least 24 h, followed by rinsing with ultrapure water.

Fish muscle: Blank and control samples were analyzed in a 10-sample batch, assuring the standards. The results were between 80% and 120%, which is the recommended range [25]. During calibration, we obtained an R^2^ > 0.999, which indicates a strong correlation of the signal. The quantification limits for the metals analyzed were as follows: Cr (0.216 mg/kg), Ni (0.016 mg/kg), Cu (0.066 mg/kg), Zn (0.712 mg/kg), As (0.007 mg/kg), Cd (0.001 mg/kg), Hg (0.01 mg/kg), and Pb (0.016 mg/kg).

Sediments: Recovery control varied between 93.9% and 116%, which is in the recommended range [25]. The linearity and strong correlation of the signal were assured, with an R^2^ > 0.999. The quantification limits for the elements were as follows: Cr (1.0 mg/kg), Ni (1.0 mg/kg), Cu (0.8 mg/kg), Zn (0.6 mg/kg), As (3.6 mg/kg), Cd (0.3 mg/kg), Hg (0.01 mg/kg), and Pb (3.0 mg/kg), Fe (2.5 mg/kg), Al (3.0 mg/kg), and Ba (0.3 mg/kg).

### 2.6. Risk Assessment

#### 2.6.1. Estimated Daily Intake (EDI)

The daily intake was calculated for each element (Cr, Ni, Cu, Zn, As, Cd, Pb, and Hg) using the following equation:

$$EDI=\frac{FIR\times Ci}{BW}$$

where FIR is the fish intake ratio (g/person/day, wet weight), Ci is the mean concentration of the element in fish muscle (mg/kg), and BW is the mean corporal weight of an adult (70 kg in this study) [27]. For adults living in rural areas, such as the ones in this study (Datém del Marañón and Loreto), the FIR was calculated for each particularly small town or village in the study, employing the data collected from the interviews, to present a more reliable measurement. This was merely to highlight that, since these small towns or villages are located far from major urban centers (1–2 days of navigating), fish intake represents a major percentage of the diet compared to other kinds of meat.

#### 2.6.2. Non-Carcinogenic Risk

The target hazard quotient (THQ) was employed to measure the non-carcinogenic risk. The THQ is the ratio of EDI to the oral reference dose (RfD, mg/kg·day); this RfD was established by the US Environmental Protection Agency (U.S. EPA 2023). This quotient was calculated as follows:

$$THQ=\frac{EDI}{RfD}$$

On this basis, there are three scenarios according to Hossain et al. (2018) [28] and Huang et al. (2019) [29]:

THQ < 1, the population will not experience any adverse health hazards.

THQ = 1, the receptors of concern may experience non-carcinogenic health effects.

THQ > 1, there is an increasing probability of adverse health hazard occurrences.

#### 2.6.3. Carcinogenic Risk (CRI)

This index is used to assess potential carcinogenic risks of fish consumption [30]. This calculation was performed only for Cr, As, and Cd, using the following equation:

$$CRI=\frac{FIR\times E_{F}\times E_{D}}{BW\times T_{A}}\times {SF}_{i}\times C_{i}$$

where E_F_ is the exposure frequency (365 day per year), ED is the exposure duration (70 years), TA is the average time (365 × ED d), and SFi is the oral slope factor (mg/kg·day) for a single element. The oral intake of carcinogenic slope factors for Cr, As, and Cd was 0.50, 0.38, and 1.50, respectively [29]. 

This brings three classifications based on the outcomes, according to [30]:

CRI < 10^−6^, the compound is safe for humans.

10^−6^ < CRI < 10^−4^, there are potential carcinogenic risks to the exposed population.

CRI > 10^−4^, the exposed people would encounter excess carcinogenic risk.

### 2.7. Statistical Analysis

Descriptive statistics was used to analyze the fish muscle and sediment concentrations. A principal component analysis (PCA) was performed to identify the relations among sample sites and the assessed elements, for both fish muscle and sediments. PCA reduces all datasets to a lower number of variables (usually the first two components) for plotting purposes. The graphical biplot correlation in the PCA was based on [31]; this analysis was performed using the free statistical software Paleontological Statistics (PAST) Version 4.03. Data analyzed in PCA were previously log-transformed; subsequently, the PCA was performed. A Spearman correlation was performed (*p* < 0.05) between fish muscle and sediments [32].

## 3. Results

### 3.1. Metal(loid)s in Fish Muscles

Regarding the element outcomes in fish (Table 3), the mean concentrations of Ni, Cu, Zn, As, Cd, and Pb demonstrated the presence of these metals at all sampling sites, with Ni, Cd, Pb, and Hg showing higher variation in at least one of the four rivers assessed. On the other hand, zinc is the metal that presented the highest mean concentrations in all four rivers, reaching a maximum of 9.88 mg/kg in the Tigre River (Table 3). Although the mean Zn concentrations were higher, they did not surpass the limits established by the FAO and ANVISA. However, the mean chromium concentrations registered in all rivers were higher than the WHO/FAO, EU, and ANVISA guidelines [27,33,34,35,36] (Table 4).

Even though the mean Hg concentrations did not surpass the guideline values in the Tigre and Pastaza rivers (0.31 and 0.28 mg/kg, respectively), the maximum values showed concentrations that are close to or even higher than the probable harmful concentration according to international guidelines. The percentage of the near and upper samples of Hg in the Tigre River was 40%, and in the Pastaza it was 16.7%.

**Figure 2:**
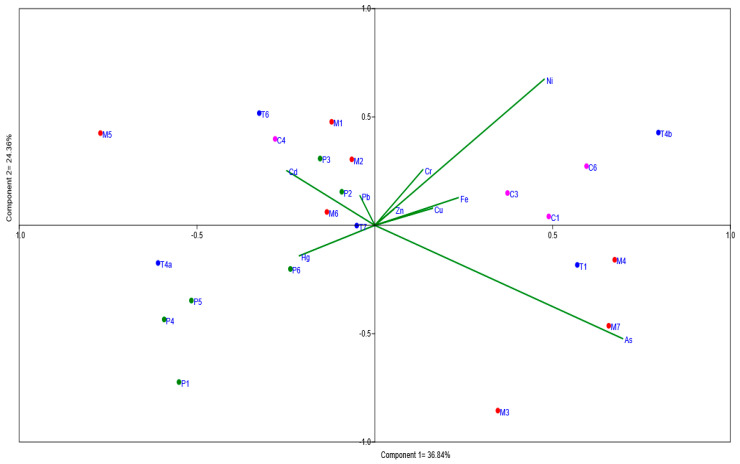
Principal component analysis of fish muscle.

The PCA for fish muscle (Figure 2) shows that there are no clear differences among the assessed rivers; however, the relationship of the sample sites to certain metals can be observed. There is a group composed of sample sites from the Pastaza (P1, P4, P5, and P6) and Tigre (T4a) rivers demonstrating a positive relation to mercury (Hg). Additionally, sample sites in the Corrientes, Tigre, and Morona rivers (C4, T6, and M5, respectively) show a positive relation to cadmium (Cd), while P2, P3, M1, and M2 (Pastaza and Morona rivers) are related to lead (Pb). Sample sites in the Morona (M3 and M7) exhibit a positive relation to arsenic (As), whereas sites in the Corrientes (C1, C3, and C6) and Tigre (T4b) show a relation to copper (Cu) and iron (Fe).

The correlation analysis among fish muscle samples indicates positive relationships between Cu and Zn, Ni and Zn, and Cr and Ni. On the other hand, there is an inverse relationship of Hg with Pb and Fe. A weak relationship between As and Cd can also be observed.

### 3.2. Sediments

Among all elements assessed in the four basins (Table 5), the highest mean concentrations of Cr, Ni, and Cu (17.85, 47.10, and 9.31 mg/kg, respectively) were observed in the Morona River. Additionally, the Morona River showed the highest mean concentration of Zn (52.19 mg/kg), with a maximum value reaching 99.80 mg/kg. In contrast, the Tigre River presented the lowest mean concentrations of Cr, Ni, Cu, and Zn (7.53, 7.00, 4.24, and 16.89 mg/kg, respectively).

**Figure 3:**
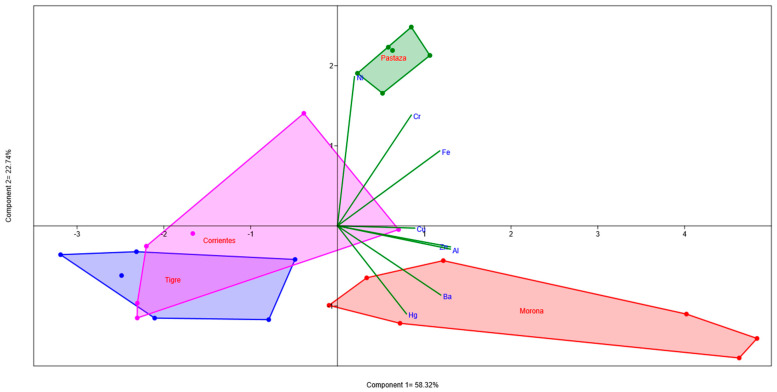
Principal component analysis for sediment.

Regarding the PCA (Figure 3), differences among the four assessed rivers were observed, with Morona River showing the clearest distinction, where the sample sites were related to Al, Ba, and Zn. The Pastaza River exhibited correlations with Ni and Cr. The Tigre and Corrientes rivers showed some similarities in certain sample sites; generally, these rivers were inversely related to Fe and Cr.

**Figure 4:**
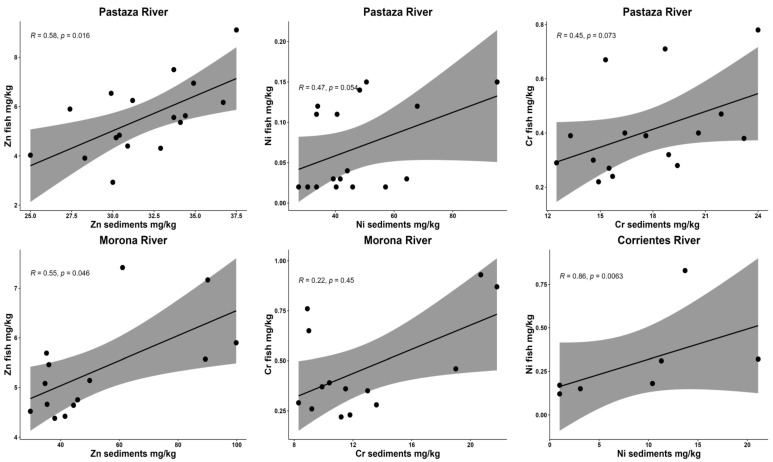
Spearman correlation analyses for sediments and fish tissues.

According to the Spearman correlation analyses performed, the metalloid contents obtained from the analyzes of fish and sediments showed significantly high ratios of Ni (Corrientes River) and Zn (Morona and Pastaza rivers), moderate ratios of Cr and Ni (Río Pastaza), and a low ratio of Cr (Río Morona) (Figure 4). 

### 3.3. Carcinogenic and Non-Carcinogenic Analysis

The oral reference doses (RfD) for Ni, Cu, Zn, Cr, As, Cd, and Hg were 20, 40, 300, 3, 0.3, 1 and 0.3 µg/kg·d, respectively [37]. Following the European Food Safety Authority’s guidelines, the RfD for Pb was set to 1.5 µg/kg·d [38].

Table 6 shows the results for RfD, EDI, THQ, and CRI for the analyzed elements in different fish muscles collected from the four river basins. Concerning THQ, Hg is the only metal that exceeds the USEPA-recommended levels in the Tigre, Morona, and Pastaza rivers (1.92, 1.83, and 1.50, respectively). When examining the CRI, Cr is the only metal that poses a potential carcinogenic risk in all four basins, with maximum values even higher in the Tigre and Morona rivers.

## 4. Discussion

The findings of this study highlight a concerning scenario regarding potential carcinogenic risks associated with mercury (Hg) and chromium (Cr) for indigenous communities in the Peruvian Amazon. The high concentrations of metals and metalloids found in the Tigre, Pastaza, and Morona rivers imply risks to public health and the well-being of communities residing in these areas. It is crucial to recognize that remote communities, situated far from urban centers, rely heavily on local resources for their livelihoods. 

However, the disruption caused by human activities directly impacts aquatic environments, leading to scenarios of bioaccumulation and biomagnification.

### 4.1. Fish Muscle Analysis

It is evident that the more remotely a community is located, the higher its fish consumption, as shown in Table 1 and Figure 1. This trend can be observed at sites like T1 and C1 in Loreto, or M2 and P2 in Datém del Marañón. The prevalence of fish in the diets of these communities is notable not only on the Peruvian side, but also in Ecuador, where it reaches up to 623 g/day in the Pastaza River [39]. Regarding the metal and metalloid concentrations in fish muscle, chromium (Cr) exceeds the established reference concentrations set out by the WHO, FAO, EU, and ANVISA in all four rivers (Table 4). Although chromium naturally originates from rocks in the basin, its presence is also linked to human activities such as petroleum extraction [40]. Additionally, the concentration of Cr can vary depending on its geological source [41], and its use in the paint industry (Cr_2_O_3_) provides chemical resistance to abrasion [42]. This type of paint is commonly used on boats in these areas.

When comparing data from the Tigre River with other global rivers (Table 7), it is evident that the maximum Cr values are higher than those observed in the Sinos and Northeast China rivers [29,43], and similar to those found in the Paraopeba [44] and Piracicaba [45] rivers in Brazil, where significant anthropogenic pollution has been reported. In contrast, the outcomes for the Tigre and Pastaza rivers are considerably lower than those observed in the Houjin River [46].

A similar pattern was observed with Cu, the levels of which were comparable to the Yangtze River [47] but much lower than the Paraopeba River. Regarding Zn, the Tigre and Pastaza rivers showed lower concentrations compared to the Paraopeba, Sinos [43], and Northeast China rivers [29], possibly due to agricultural activities. Mercury in the Tigre and Pastaza Rivers presents maximum concentrations that exceed the values reported in the Northeast China [29] and Gomti [28] Rivers. In the case of Peru, fish in other Amazonian rivers have registered higher values than the FEPA and WHO guidelines [50].

### 4.2. Sediment Analysis

The outcomes indicate that the Cr, Ni, and Zn concentrations surpass the NOAA, ISQG, and EPA international guidelines (Table 5). According to the statistical analysis, significant correlations between sediment and fish muscle were identified for Ni, Zn, and Cr (Figure 4). This phenomenon could be related to bioaccumulation occurrences, particularly noticeable in the case of Cr. 

### 4.3. Human Health Risks

The findings of this study highlight a concerning scenario regarding possible carcinogenic risks related to mercury (Hg) and chromium (Cr) for indigenous communities in the Peruvian Amazon. The recommended daily intake limits for metals [33,51] are 0.2 mg/day for Cr, 1.5 mg/day for Cu, 0.002 mg/day for As, 0.007 mg/day for Cd, and 0.0001 mg/day for Hg per person. It is evident from the findings that the EDI calculated for metals (except Hg) in this study did not exceed the recommended values. However, the Hg concentrations in the Tigre River (0.00057 mg/kg·person), Morona River (0.00055 mg/kg·person), and Pastaza River (0.00045 mg/kg·person) are 5 to 6 times higher than the recommended values.

Regarding the risks to human health, it is noteworthy that, among the areas assessed in this study, three basins are more likely to pose negative health effects due to high THQ values for Hg and Cr. Sampling sites such as T4, M1, M3, and P3 have THQ values above 1 for Hg, while T1, T6, M2, P2, and P4 reach values of almost 3, indicating an increased probability of adverse health effects. Of particular concern is sampling site M3, which reports a THQ of 6.43, indicating a substantial potential health impact. Similar studies have also shown high THQ values in Amazonian fish species, ranging from 1.04 to 4.18 [52,53], categorizing them as unsuitable for human consumption.

Regarding the mean THQ value for Cr, it did not exceed the reference value in any of the four basins. However, it is important to note that M1 and M2 registered 1.18 and 1.44, respectively. This is a health alert for the Morona River Basin, as well as for the Shapra and Wampis indigenous communities, which should be continuously monitored by the appropriate authorities. Additionally, the CRI values for Cr were higher than the reference values in all four basins, indicating that fish consumption may expose people to excess carcinogenic risk. The Morona River Basin shows the highest CRI values, with sites like M1, M2, and M4 having CRI values of 0.0018, 0.0022, and 0.001, respectively.

In the case of Hg, THQ’s potential risks to the environment are probably the result of human activities developed in the watershed, which includes Ecuadorian territory. In Peru, there are no official reports of legal gold exploitation in the three basins; however, a metallogenic gold map was recently published in Peru [54]. This map clearly establishes that certain Amazonian rivers (Santiago River, the Marañón, the middle part of the Morona River, and the lower part of the Pastaza River are favorable for placer, alluvial, and moraine-type gold deposits. Therefore, the possible presence of informal or illegal dredgers furtively exploiting gold with unsafe techniques and acting as potential sources of Hg in these rivers cannot be ruled out. On the other hand, studies indicate the presence of mining activities, mainly illegal gold mining, including the upper basin of the Morona and Pastaza rivers [11,12,55].

## 5. Conclusions

The sediment and fish muscle assessment in the four rivers revealed the actual concentrations of metals in the environment. In the case of sediments, Cr, Ni, and Zn exceeded the concentrations of international standards (NOAAA and ISQG). Similarly, high concentrations of Cr and Hg were found in fish muscle in the Tigre, Morona, and Pastaza rivers.

On the other hand, the non-carcinogenic risk analysis indicates that Hg is considered to be a health threat, especially in the Tigre, Morona, and Pastaza rivers, which means that there is an increasing likelihood of adverse health events related to this metal. Meanwhile, the carcinogenic risk assessment shows that Cr is a potential driver in all four rivers, so exposed people would face an excess carcinogenic risk due to this metal. Thus, the evidence found regarding the high concentrations of metals (Hg, Cr) could become chronic accumulation in human organisms, resulting in a public health problem.

The results presented in this study provide information that will serve as a basis for decision-making in future management plans that would help maintain or restore the riparian environment in the Department of Loreto.

## Figures and Tables

**Table 1:** Sampling sites, geographical information, and daily fish intake for the Corrientes, Tigre, Morona, and Pastaza rivers. Data on daily fish consumption were obtained through interviews with residents.

Site	Small Town or Village	No. of Fish Specimens per Pool	River	District/Province	Human Daily Fish Intake (g/Person/Day)
C1	Nueva Valencia	18	Corrientes	Trompeteros/Loreto	103.00
C3	Pucacuro	19	Corrientes	Trompeteros/Loreto	103.23
C4	Trompeteros	17	Corrientes	Trompeteros/Loreto	82.94
C6	Providencia	31	Corrientes	Trompeteros/Loreto	60.00
T1	Paiche Playa	16	Tigre	Tigre/Loreto	146.14
T4a	Intuto	19	Tigre	Tigre/Loreto	122.45
T4b	28 de Julio	28	Tigre	Tigre/Loreto	175.68
T6	Piura	11	Tigre	Tigre/Loreto	134.46
T7	Nueva York	30	Tigre	Tigre/Loreto	56.62
M1	Inka Roca-Tigreyacu	10	Morona	Morona/Datém del Marañón	302.36
M2	Caballito-Tipishcacocha	19	Morona	Morona/Datém del Marañón	383.00
M3	Shoroya nuevo	10	Morona	Morona/Datém del Marañón	382.11
M4	Pinshacocha	17	Morona	Morona/Datém del Marañón	63.01
M5	San Martin	41	Morona	Morona/Datém del Marañón	62.53
M6	Puerto Alegría	13	Morona	Morona/Datém del Marañón	63.15
M7	Puerto América	15	Morona	Morona/Datém del Marañón	64.24
P1	Nuevo Soplín	13	Pastaza	Morona/Datém del Marañón	60.00
P2	Loboyacu	18	Pastaza	Morona/Datém del Marañón	302.37
P3	Sungache	18	Pastaza	Morona/Datém del Marañón	100.00
P4	San Fernando	10	Pastaza	Morona/Datém del Marañón	101.20
P5	Musakarusha	12	Pastaza	Morona/Datém del Marañón	82.41
P6	Nueva Alianza	8	Pastaza	Morona/Datém del Marañón	84.43

**Table 2:** Basic biological information of fish collected from the four Amazonian rivers in this study.

Scientific Name	Name	River				Total	Feeding Regime	Habitat
		Corrientes	Tigre	Morona	Pastaza			
*Acestrorhynchus falcirostris*	Big-eyed cachorro	1	2		1	4	Piscivorous	Benthopelagic
*Adontosternarchus balaenops*	Ghost knifefish		4			4	Carnivorous	Benthopelagic
*Ageneiosus inermis*	Manduba	1		2	1	4	Piscivorous	Pelagic
*Amblydoras affinis*	Rego rego			7		7	Omnivore	Demersal
*Ancistrus dolichopterus*	Bushymouth catfish			1		1	Omnivore	Demersal
*Ancistrus temminckii*	Carachama			4		4	Omnivore	Demersal
*Anodus elongatus*	Yulilla	1	3			4	Omnivore	Pelagic
*Astronatus ocellatus*	Oscar				2	2	Omnivore	Benthopelagic
*Biotodoma cupido*	Greenstreaked eartheater			1	2	3	Omnivore	Benthopelagic
*Brachyplatytoma vaillantii*	Laulao catfish				1	1	Piscivorous	Demersal
*Brycon amazonicus*	Sábalo		1	3		4	Omnivore	Benthopelagic
*Bunocephalus coraoideus*	Banjo catfish			1		1	Omnivore	Demersal
*Calophysus macropterus*	Zamurito				1	1	Carnivorous	Demersal
*Chaetobranchus flavescens*	Bujurqui vaso		15	1	5	21	Omnivore	Benthopelagic
*Cheirodon interruptus*	Uruguay tetra		2			2	Omnivore	Benthopelagic
*Chilodus punctatus*	Spotted headstander		5			5	Omnivore	Pelagic
*Cichla monoculus*	Peacock bass	1			2	3	Carnivorous	Benthopelagic
*Cichla ocellaris*	Peacock cichlid		1			1	Carnivorous	Benthopelagic
*Corydoras arcuatus*	Skunk corydoras			1		1	Detritivore	Demersal
*Curimatella meyeri*	Yahuarachi				1	1	Detritivore	Benthopelagic
*Cynodon gibbus*	Chambira				1	1	Carnivorous	Pelagic
*Hemiodus microlepis*	Yulilla				1	1	Omnivore	Benthopelagic
*Hemisorubim platyrhynchos*	Porthole shovelnose catfish	1				1	Carnivorous	Demersal
*Heros efasciatus*	Bujurqui acha vieja				1	1	Omnivore	Benthopelagic
*Heros severus*	Banded cichlid			1		1	Omnivore	Benthopelagic
*Hoplerythrinus unitaeniatus*	Aimara		2	1	9	12	Carnivorous	Pelagic
*Hoplias malabaricus*	Trahira	3	5	7	7	22	Carnivorous	Benthopelagic
*Hoplosternum littorale*	Atipa				4	4	Detritivore	Demersal
*Hyphessobrycon copelandi*	Wira mojara			5		5	Omnivore	Benthopelagic
*Hypophthalmus edentatus*	Highwaterman catfish	16	1			17	Herbivore	Pelagic
*Hypselecara temporalis*	Emerald cichlid				5	5	Omnivore	Benthopelagic
*Lamontichthys stibaros*	Shitari	1				1	Detritivore	Demersal
*Leporinus friderici*	Threespot leporinus			6		6	Omnivore	Benthopelagic
*Megaleporinus trifasciatus*	Lisa	1	1			2	Omnivore	Benthopelagic
*Myleus rubripinnis*	Redhook myleus	10	7	1	6	24	Omnivore	Benthopelagic
*Pimelodus blochii*	Bloch’s catfish	1	2	1		4	Omnivore	Benthopelagic
*Pinirampus pirinampu*	Flatwhiskered catfish	1				1	Carnivorous	Demersal
*Potamorhina altamazonica*	Yahuarachi	6	22	5	4	37	Detritivore	Benthopelagic
*Potamotrygon magdalenae*	Magdalena river stingray		2			2	Detritivore	Benthopelagic
*Prochilodus nigricans*	Black prochilodus	1	11	21	12	45	Detritivore	Benthopelagic
*Psalidodon fasciatus*	Banded astyanax			1		1	Omnivore	Benthopelagic
*Psectrogaster amazonica*	Chio Chio	11	26	4		41	Detritivore	Benthopelagic
*Pseudorinelepis genibarbis*	Carachama negra		1			1	Detritivore	Benthopelagic
*Pterodoras granulosus*	Granulated catfish		2	2		4	Omnivore	Demersal
*Pterygoplichthys pardalis*	Amazon sailfin catfish			16		16	Detritivore	Demersal
*Pygocentrus nattereri*	Red piranha			4	1	5	Carnivorous	Pelagic
*Rhaphiodon vulpinus*	Biara			1		1	Carnivorous	Pelagic
*Rhytiodus microlepis*	Lisa			1		1	Herbivorous	Benthopelagic
*Roeboides myersii*	Dentón	2	1	4	1	8	Carnivorous	Benthopelagic
*Satanoperca jurupari*	Demon eartheater				5	5	Carnivorous	Benthopelagic
*Schizodon fasciatus*	Characin			5	4	9	Omnivore	Benthopelagic
*Semaprochilodus insignis*	Kissing prochilodus	1	2		2	5	Detritivore	Benthopelagic
*Serrasalmus rhombeus*	Red-eye piranha	1		10	3	14	Carnivorous	Benthopelagic
*Squaliforma emarginata*	Carachama blanca			2		2	Detritivore	Demersal
*Trachelyopterus galeatus*	Novia cunchi	2		2	4	8	Omnivore	Demersal
*Triportheus angulatus*	Sardina	3	3	4	7	17	Omnivore	Benthopelagic

**Table 3:** Metal(loid)s’ descriptive statistics in fish muscle (mg/kg wet weight); mean, standard deviation, range, and coefficient of variation (expressed in %) for the four rivers assessed.

Element	Tigre (n = 104)			Morona (n = 125)			Corrientes (n = 85)			Pastaza (n = 79)		
	Mean ± SD	Range	CV	Mean ± SD	Range	CV	Mean ± SD	Range	CV	Mean ± SD	Range	CV
Cr	0.73 ± 0.34	0.32–1.29	51.95	0.46 ± 0.24	0.23–0.93	53.2	0.53 ± 0.10	0.43–0.67	19.01	0.40 ± 0.16	0.29–0.72	40.88
Ni	0.22 ± 0.19	0.04–0.64	94.74	0.10 ± 0.03	0.04–0.13	32.07	0.19 ± 0.09	0.08–0.32	45.8	0.06 ± 0.05	0.02–0.13	92.2
Cu	0.54 ± 0.19	0.22–0.89	33.36	0.46 ± 0.13	0.22–0.67	28.89	0.47 ± 0.11	0.32–0.68	24.31	0.41 ± 0.13	0.28–0.59	32.74
Zn	6.30 ± 1.76	3.91–9.88	25.19	5.34 ± 0.96	4.37–7.42	15.79	6.58 ± 0.85	5.60–7.64	12.98	5.49 ± 1.32	4.17–7.72	24.03
As	0.02 ± 0.02	ND–0.07	109.67	0.02 ± 0.02	ND–0.07	104.79	0.02 ± 0.01	ND–0.03	60.61	0.01 ± 0.002	ND–0.01	21.02
Cd	0.006 ± 0.002	0.002–0.01	40.53	0.006 ± 0.01	0.001–0.02	94.68	0.005 ± 0.002	0.003–0.01	31.11	0.01 ± 0.004	0.004–0.01	32.6
Pb	0.05 ± 0.02	0.03–0.07	31.91	0.04 ± 0.02	0.01–0.07	54.98	0.06 ± 0.04	0.02–0.12	70.6	0.06 ± 0.03	0.03–0.13	53.29
Hg	0.31 ± 0.10	0.20–0.43	33.43	0.18 ± 0.10	0.10–0.39	54.9	0.11 ± 0.01	0.10–0.13	11.24	0.28 ± 0.14	0.12–0.52	50.02

ND = not detected; n: the number of samples of fish muscle.

**Table 4:** Limit concentrations of metals in fish muscle according to international guidelines (mg/kg).

Guideline/Reference	Cr	Ni	Cu	Pb	Zn	Hg	As	Cd
FAO [27]				0.2	50	0.5		0.05
FAO/WHO [33]	0.05	8.97	30	0.05				0.015
China QS [34]	2.0			0.5		0.5	0.1	
European Union [35]	0.5			0.3		0.5		0.05
ANVISA [36]	0.1	5.0	30		50	0.5	0.5	

**Table 5:** Descriptive statistical data of the metal(loid)s found in sediments. Standard deviation and range (min–max) values in mg/kg, and coefficient of variation (CV) expressed in %.

Element	Tigre (n = 12)			Morona (n = 14)			Corrientes (n = 12)			Pastaza (n = 12)			Guidelines		
	Mean ± SD	Range	CV	Mean ± SD	Range	CV	Mean ± SD	Range	CV	Mean ± SD	Range	CV	NOAAA	ISQG	SQG-EPA
Cr	7.53 ± 1.77	1.90–16.00	63.34	12.74 ± 1.51	8.30–21.80	35.43	11.58 ± 2.23	3.20–35.30	79.65	17.85 ± 3.06	12.50–24.00	22.77	13	37.2	43.4
Ni	7 ± 1.28	3.60–10.40	32.63	10.83 ± 2.39	7.20- 18.0	31.28	10.57 ± 5.77	3.10–21.10	54.59	47.10 ± 8.67	27.5–95.10	39.62	9.9	18	22.7
Cu	4.24 ± 0.49	2.20–8.40	58.67	7.48 ± 1.25	1.90–21.00	96.95	4.58 ± 1.98	2.10–8.70	65.05	9.31 ± 1.95	5.80–18.00	42.42	25	35.7	31.6
Zn	16.89 ± 2.52	5.40–38.40	62.26	52.19 ± 13.58	29.70–99.80	45.18	20.78 ± 2.32	8.90–37.60	44.86	31.94 ± 3.19	25.0–39.50	13.13	38	123	121
Al	2523.78 ± 378.2	626.6–7447.0	79.45	9489.71 ± 1239.4	4467.0–21,160.0	62.98	2988.03 ± 206.5	941.4–7365	63.71	5714.92 ± 379.2	3404.0–8674.0	25.76	2600		
Fe	5671.50 ± 424.1	1911.0–12,585.0	62.94	14,114.14 ± 2921.6	8664.0–23,352.0	34.65	7160.2 ± 322.2	2366–14,375	56.69	15,543.0 ± 1077.5	12,993.0–19,773.0	12.99	18,000		20,000
Ba	26.80 ± 2.1	8.60–71.8	71.48	89.93 ± 11.55	52.7–158.1	38.86	35.9 ± 2.88	16.9–51.7	35.88	34.97 ± 7.1	19.40–47.70	22.58	0.70		
As	<3.6	0	0.00	<3.6	0	0	<3.6	0	0	<3.6	0	0	1.1	5.9	<3
Cd	<0.3	0	0.00	<0.3	0	0	<0.3	0	0	<0.3	0	0	0.3	0.6	0.99
Pb	<3.0	0	0.00	<3.0	0	0	<3.0	0	0	<3.0	0	0	17	35	35.8
Hg	0.02 ± 0.01	0.01–0.06	70.46	0.04 ± 0.02	0.01–0.07	55.22	0.02 ± 0.01	0.01–0.03	50.65	0.02 ±0.01	0.01–0.04	83.85	0.05	0.17	0.18

n: the number of samples of sediment. NOAA: National Oceanic and Atmospheric Administration (USA). ISQG: Interim Sediment Quality Guidelines (Canada). SQG-EPA: Small Quantity Generator U.S. Environmental Protection Agency (USA).

**Table 6:** Carcinogenic and non-carcinogenic risk analysis of elements in fish consumed in Loreto, and carcinogenic risk index for Cr (10^−4^), As, and Cd (10^−6^). RfDs (µg/kg·day), CRI range (min–max).

Elements	RfDs	Tigre			Morona			Corrientes			Pastaza		
		EDI	THQ	CRI	EDI	THQ	CRI	EDI	THQ	CRI	EDI	THQ	CRI
Cr	1	1.33	0.44	6.6 (1.3–13.1)	1.58	0.53	7.8 (1.01–21.5)	0.68	0.23	3.3 (1.8–4.8)	0.72	0.24	3.5 (1.3–9)
Ni	20	0.40	0.02		0.26	0.01		0.22	0.01		0.14	0.01	
Cu	40	0.97	0.02		1.38	0.03		0.57	0.01		0.67	0.02	
Zn	300	11.31	0.04		14.66	0.05		8.11	0.03		9.08	0.03	
As	* 0.3	0.05	0.16	71.6 (8.6–183.8)	0.05	0.17	78.4 (2.99–312.9)	0.02	0.08	35.5 (5.47–64.4)	0.02	0.07	30.3 (14.9–85.2)
Cd	1	0.01	0.01	4.6 (0.7–7.6)	0.01	0.01	4.8 (0.8–14.6)	0.01	0.01	2.2 (1.7–2.7)	0.02	0.02	8.2 (1.4–24.1)
Pb	1.5	0.09	0.06		0.10	0.07		0.09	0.06		0.11	0.07	
Hg	** 0.3	0.57	1.92		0.55	1.83		0.14	0.45		0.45	1.50	

* Inorganic arsenic; ** elemental mercury, and mercury compounds.

**Table 7:** Comparison of metal(loid) concentrations (mg/kg ww) in fish among different riverine environments.

River	Country	Cr	Ni	Cu	Zn	As	Cd	Pb	Hg	References
Tigre	Peru	0.32–1.29	0.04–0.49	0.28–0.75	4.14–8.28	0.003–0.06	0.002–0.01	0.03–0.07	0.20–0.41	This study
Pastaza	Peru	0.29–0.72	0.02–0.13	0.28–0.59	4.17–7.72	ND–0.01	0.004–0.01	0.03–0.13	0.12–0.52	This study
Abaeté and Paraopeba	Brazil	0.42–1.7		5.62–32.2	8.88–21.1		0.02–0.33	0.17–2.11		[44]
Piracicaba	Brazil	0.05- 1.11	0.05–2.83	0.03–4.18		0.73–0.93	0.02–0.92	0.66–3.25		[45]
Sinos	Brazil	0.09–0.38	-	1.43–5.4	22.9–34.6	1.47–8.79	0.07–0.8	1.2–2.5	-	[43]
Northeast of China	China	ND–0.49	ND–0.78	0.067–0.97	2.49–51.38	ND–0.39	ND–0.009	ND–0.70	ND–0.82	[29]
Yangtze	China	0.10–0.24	-	0.77–1.22	2.8–7.55	-	0.046–0.12	0.21–0.81	-	[47]
Houjin	Taiwan	ND–83.44	ND–34.59	ND–380	ND–107	ND–3.11	ND–14.5	ND–13.5	ND–0.38	[46]
Gomti	Bangladesh	0.004	-	-	-	0.002	BDL	BDL	0.006	[28]
Alto Solimões	Brazil								0.081–0.33	[48]
Rio Amazonas–Manaos	Brazil								0.0–2.18	[49]
Bajo Napo–Loreto	Perú								0.04–1.94	[50]

ND: not detected; BDL: below detectable level.

## Data Availability

Data available on request due to ethical restrictions.
